# GSK-3β Overexpression Alters the Dendritic Spines of Developmentally Generated Granule Neurons in the Mouse Hippocampal Dentate Gyrus

**DOI:** 10.3389/fnana.2017.00018

**Published:** 2017-03-10

**Authors:** Noemí Pallas-Bazarra, Asta Kastanauskaite, Jesús Avila, Javier DeFelipe, María Llorens-Martín

**Affiliations:** ^1^Centro de Biología Molecular Severo Ochoa – Consejo Superior de Investigaciones Cientificas, Universidad Autónoma de MadridMadrid, Spain; ^2^Centro de Investigación Biomédica en Red sobre Enfermedades NeurodegenerativasMadrid, Spain; ^3^Cajal Laboratory of Cortical Circuits, Centro de Tecnologá Biomédica, Universidad Politécnica de MadridMadrid, Spain; ^4^Cajal Institute – Consejo Superior de Investigaciones CientificasMadrid, Spain; ^5^Department of Molecular Biology, Faculty of Sciences, Universidad Autónoma de MadridMadrid, Spain

**Keywords:** GSK-3β, hippocampus, dentate gyrus, granule neurons, dendritic spines, intracellular injection

## Abstract

The dentate gyrus (DG) plays a crucial role in hippocampal-related memory. The most abundant cellular type in the DG, namely granule neurons, are developmentally generated around postnatal day P6 in mice. Moreover, a unique feature of the DG is the occurrence of adult hippocampal neurogenesis, a process that gives rise to newborn granule neurons throughout life. Adult-born and developmentally generated granule neurons share some maturational aspects but differ in others, such as in their positioning within the granule cell layer. Adult hippocampal neurogenesis encompasses a series of plastic changes that modify the function of the hippocampal trisynaptic network. In this regard, it is known that glycogen synthase kinase 3β (GSK-3β) regulates both synaptic plasticity and memory. By using a transgenic mouse overexpressing GSK-3β in hippocampal neurons, we previously demonstrated that the overexpression of this kinase has deleterious effects on the maturation of newborn granule neurons. In the present study, we addressed the effects of GSK-3β overexpression on the morphology and number of dendritic spines of developmentally generated granule neurons. To this end, we performed intracellular injections of Lucifer Yellow in developmentally generated granule neurons of wild-type and GSK-3β-overexpressing mice and analyzed the number and morphologies of dendritic spines (namely, stubby, thin and mushroom). GSK-3β overexpression led to a general reduction in the number of dendritic spines. In addition, it caused a slight reduction in the percentage, head diameter and length of thin spines, whereas the head diameter of mushroom spines was increased.

## Introduction

The hippocampal dentate gyrus (DG) plays a pivotal role in learning and episodic memory (Garthe et al., 2009; Deng et al., 2010; Sahay et al., 2011). In addition, it is one of the few brain regions in which neurogenesis occurs during adulthood (Bayer and Altman, 1975; Kempermann et al., 2015). The vast majority of neurons present in the DG are excitatory granule neurons, which participate in both classic (Amaral, 1978) and alternative (Kohara et al., 2014; Llorens-Martin et al., 2015a) trisynaptic hippocampal circuits. Although still a matter of debate, the function of newborn and developmentally generated granule neurons appears to differ (Nakashiba et al., 2012). The main afferent connection of granule neurons is the perforant pathway, which arises in the entorhinal cortex (Amaral and Witter, 1989), although other cortical and non-cortical structures innervate them (Wang et al., 2005; Vivar et al., 2012; Deshpande et al., 2013; Kohara et al., 2014). The most predominant innervation from the perforant pathway occurs in the two outer thirds of the molecular layer, where the dendrites of granule neurons are located (Amaral and Witter, 1989). Importantly, adult-born and developmentally generated granule neurons differ, among other aspects, in their positioning within the granule cell layer. In this regard, the former are located exclusively in the inner third of this layer, whereas the latter are found in the two outer thirds (Kempermann et al., 2003, 2004).

The formation of new afferent excitatory synaptic contacts in granule neurons takes place in dendritic spines (for simplicity, spines) (Krzisch et al., 2015; Herms and Dorostkar, 2016). Of note, the morphology of these spines is quite variable. In general terms, they can be classified into the following three categories on the basis of morphological criteria: stubby, thin, and mushroom. Interestingly, the morphology and volume of spines correlate with the dynamics (plasticity) and strength of the synaptic contacts (Matsuzaki et al., 2001; Rochefort and Konnerth, 2012; Heck and Benavides-Piccione, 2015; Segal, 2016), which, in turn, are related to long-term potentiation (LTP) (Bliss and Collingridge, 1993). In this regard, a key protein that regulates LTP is glycogen synthase kinase 3 (GSK-3) (Hooper et al., 2007; Peineau et al., 2007). GSK-3 refers to two isoforms, namely GSK-3α and GSK-3β (Woodgett, 1990). In the central nervous system (CNS), GSK-3β plays a pivotal role in memory regulation (Zhu et al., 2007; Avila et al., 2010; Kettunen et al., 2015; Pardo et al., 2016). In line with this, a transgenic mouse overexpressing GSK-3β in excitatory neurons of the forebrain was generated in our laboratory (Lucas et al., 2001). Noteworthy, the overexpression of GSK-3β was particularly high in the DG of these animals (Fuster-Matanzo et al., 2011; Sirerol-Piquer et al., 2011). Accordingly, this animal model exhibited marked hippocampal-dependent memory impairment (Hernandez et al., 2002). In addition, it has recently been shown that GSK-3β overexpression profoundly impairs connectivity (e.g., in the number and volume of the postsynaptic densities and spines) of adult-born granule neurons (Llorens-Martin et al., 2013, 2016). However, the putative effects of GSK-3β overexpression on the structural plasticity of developmentally generated granule neurons have not been addressed to date. In this regard, here we analyzed the density and morphology of the spines of these neurons. With this aim, we labeled individual granular cells by performing intracellular injections of Lucifer Yellow in fixed brain tissue. To ensure their developmental origin, only granule neurons located in the outer third of the granule cell layer received the injections. Our results revealed that GSK-3β overexpression alters the density and morphology of spines of granule neurons of developmental origin.

## Materials and Methods

### Animals

Transgenic mice overexpressing GSK-3β under the control of the CamKII promoter were generated as previously described (Lucas et al., 2001). Briefly, GSK-3β- overexpressing (GSK3-OE) mice were bred by crossing TetO mice (carrying the bidirectional tet-responsive promoter followed by the GSK-3β and β-galactosidase cDNAs, one in each direction) with CamKIIα-tTA mice. Wild-type (WT) littermates resulting from the crossing CamKIIα-tTA mice (heterozygous) with TetO-GSK-3β (heterozygous) ones were used as controls. A marked increase in GSK-3β levels was evident in various regions of the forebrain of GSK3-OE mice. In particular, high expression was observed in the hippocampus (Llorens-Martin et al., 2013). Animals were housed in a specific pathogen-free colony facility at the *Centro de Biología Molecular “Severo Ochoa”* in accordance with European Community Guidelines (directive 86/609/EEC) and were handled following European and local animal care protocols. All the protocols were approved by the local (*Centro de Biología Molecular “Severo Ochoa”*, AEEC-CBMSO-23/172) and national (Comunidad de Madrid, PROEX 205/15) Welfare Animal Ethics Committees. Four female mice (5 months old) per genotype were used. Animals were housed in groups of four per cage. Mice from both genotypes were randomly assigned to each cage in order to ensure a homogenous distribution of genotypes.

### Sacrifice and Tissue Sectioning

Mice were fully anesthetized with an intraperitoneal pentobarbital injection (EutaLender, 60 mg/kg bw) and transcardially perfused with saline followed by 4% paraformaldehyde in 0.1 M phosphate buffer (pH = 7.4). Brains were quickly removed and post-fixed overnight in the same fixative at 4°C. The next day, they were washed three times with cold 0.1 M phosphate buffer. Coronal vibratome sections were obtained (200 μm: Lancer 1000; St Louis, MO, USA) and then processed for intracellular injections, as previously described (Buhl and Schlote, 1987; Einstein, 1988; Elston et al., 1997).

### Intracellular Injections of Lucifer Yellow

Sections were prelabeled with 4,6-diamidino-2-phenylindole (DAPI) in 0.1 M phosphate buffer pH 7.4 for 1–2 min (D9542; Sigma, St. Louis, MO, USA). A continuous current was then used to inject individual cells with Lucifer yellow dye (8% in 0.1 M Tris buffer, pH 7.4 (L0259; Sigma, St. Louis, MO, USA)). At least 30 individual granule neurons in the DG per animal were injected. Intracellular injections were performed in a semiregular array ≈30 μm from the surface (in the second focal plane of DAPI-stained nuclei). Cells were injected until all the individual dendrites could be traced to an abrupt end at their distal tips, thereby indicating that the dendrites were completely filled and ensuring that the fluorescence did not diminish at a distance from the soma. In order to ensure the developmental origin of the neurons, only the outermost line of cell nucleus was used to perform the intracellular injections (Kempermann et al., 2003). In order to rule out the possibility that differences in the migration of newborn granule neurons may bias our observations, we quantified the distance migrated by retrovirally labeled fully mature newborn granule neurons of 10 weeks of age in animals of both genotypes. It should be noted that newborn granule neurons are considered fully mature at 8 weeks of cell age and onwards. The migration of 100 cells per genotype was analyzed and it is shown in **Supplementary Figure S1**.

### Retroviral Stock Preparation

We used a retroviral stock encoding for GFP (Zhao et al., 2006). The plasmids used to produce the virus were kindly provided by Prof. FH. Gage. Retroviral stocks were concentrated to working titers of 1 × 10^7^ – 2 × 10^8^ pfu/ml by ultracentrifugation (Zhao et al., 2006). Since the retroviruses used are engineered to be replication-incompetent, only dividing cells at the time of surgery can be infected (Zhao et al., 2006).

### Stereotaxic Surgery

Mice were anesthetized with Isoflourane and placed in a stereotaxic frame. Coordinates (mm) relative to bregma in the anteroposterior, mediolateral, and dorsoventral axes were as follows: dentate gyrus (DG) [–2.0, 1.4, 2.2]. 2 μl/DG of virus solution was infused at a rate of 0.2 μl/min via a glass micropipette. Animals were 8 weeks old at the time of retroviral injections.

### Immunostaining

After neuron injections, the sections were double immunostained with a rabbit anti-Lucifer yellow antibody [generated at the Cajal Institute (Benavides-Piccione et al., 2013; Merino-Serrais et al., 2013) 1:400000] and a mouse anti β-Galactosidase antibody (Promega 1:3000) in blocking solution (2% BSA (A3425; Sigma), 1% Triton X-100 (30632; BDH Chemicals), and 5% sucrose in 0.1 M phosphate buffer). The anti-Lucifer yellow antibody was used in order to amplify the Lucifer yellow signal and to avoid fading during confocal image acquisition. To detect the binding of primary antibodies, the following secondary antibodies were used: a biotinylated donkey anti-rabbit secondary antibody (1:200, RPN1004; Amersham Pharmacia Biotech) followed by a streptavidin conjugated with Alexa 488 (1:1000; Molecular Probes, Eugene, OR, USA); and a goat anti-mouse secondary antibody conjugated with Alexa 594 (1:1000; Molecular Probes, Eugene, OR, USA). The sections were then washed and mounted with ProLong Gold Antifade Reagent (Invitrogen Corporation, Carlsbad, CA, USA).

In order to measure the distance migrated by newborn granule neurons, immunostaining against GFP was performed. For immunohistochemical analysis, series of 50- μm thick brain slices were made up randomly of one section from every 9th. Slices were initially pre-incubated in phosphate buffer with 1 % Triton X-100 and 1% bovine serum albumin, and then immunohistochemistry was performed as described previously (Pallas-Bazarra et al., 2016). A rabbit anti-GFP (Invitrogen 1:1000) and a secondary donkey anti-rabbit Alexa 555-conjugated antibody (Molecular Probes 1:1000) were used.

All sections were counterstained with DAPI (Calbiochem, 1:5000). The following incubation periods were used: 48 h at 4°C for primary antibodies; 24 h at 4°C for secondary antibodies; and 10 min for DAPI incubation.

### Migration into the GL

In order to measure the migration of retrovirally labeled 10-week-old newborn granule neurons, the distance between the hilar border of the subgranular zone (SGZ) and the center of the cell nucleus of each GFP+ cell was measured. Hundred cells per genotype were analyzed. Data are presented as mean ± SEM.

### Spine Reconstruction and Morphometric Analysis

Images for spine analysis were acquired with a Zeiss confocal microscope (LSM 710, Carl Zeiss MicroImaging GmbH, Germany) using a 63x oil objective. Three-dimension Z-stacks (voxel size 0.057 μm × 0.057 μm × 0.14 μm) were obtained in order to reconstruct entire dendrites of granule cells of WT and GSK3-OE mice.

For the analysis of spine density and volume, a minimum of 40 individual whole branches per genotype were reconstructed and subdivided into 10-μm segments using Imaris software x 64 7.6.4. Thus, the number and volume of spines were calculated on the basis of their position (distance from the soma) along the dendrite. For the analysis of spine morphology, a minimum of 40 randomly chosen dendritic fragments per genotype were reconstructed using *NeuronStudio* software. After that, the spines were detected by the software and assigned to one of three categories, namely stubby, thin, or mushroom. *NeuronStudio* software classifies the spines according to their head to neck diameter ratio, length to head diameter ratio and head diameter. Critical values that control this classification scheme can be adjusted (Rodriguez et al., 2008). Thus, we applied the following parameters for classification purposes: neck ratio (head to neck diameter ratio) 0.900 pixels; thin ratio (length to head diameter ratio) 2.500 pixels; and mushroom size (head diameter) 0.450 μm. Each spine was checked manually in order to ensure accurate classification. The head spine diameter, and the approximate measure of spine length (Max-DTS) were calculated for each type of spine. In addition, the percentage of each type of spines was calculated. The morphometric parameters provided by NeuronStudio software were as follows:

HEAD-DIAMETER: The diameter of the head of the spine.

MAX-DTS: The distance from the “tip” of the spine to the surface of the model. This value is therefore an approximate measure of the length of the spine. Note that the tip is the voxel contained within the spine that is furthest from the surface.

### Statistical Analysis

Statistical analysis was performed using the SPSS 23 software (SPSS, 1989; Apache Software Foundation, Chicago, IL, USA). The Kolmogorov–Smirnov test was used to test the normality of the sample distribution. For the analysis of the dendritic spine number and volume and of the head diameter and Max-DTS of the spines, data were analyzed by a Student’s *t*-test in the case of normal sample distribution, or by a nonparametric test (Mann–Whitney *U*-test) in those cases in which normality could not be assumed. Graphs represent mean values ± SEM. The distributions of dendritic spine sizes, head spine diameters and Max-DTS were compared by means of a Kolmogorov–Smirnov *Z* test. The analysis of the percentage of the different types of spine was accomplished by a chi-squared (χ2) test.

## Results

### GSK-3β Overexpression Decreases the Spine Density of Developmentally Generated Granule Neurons *In vivo*

**FIGURE 1:**
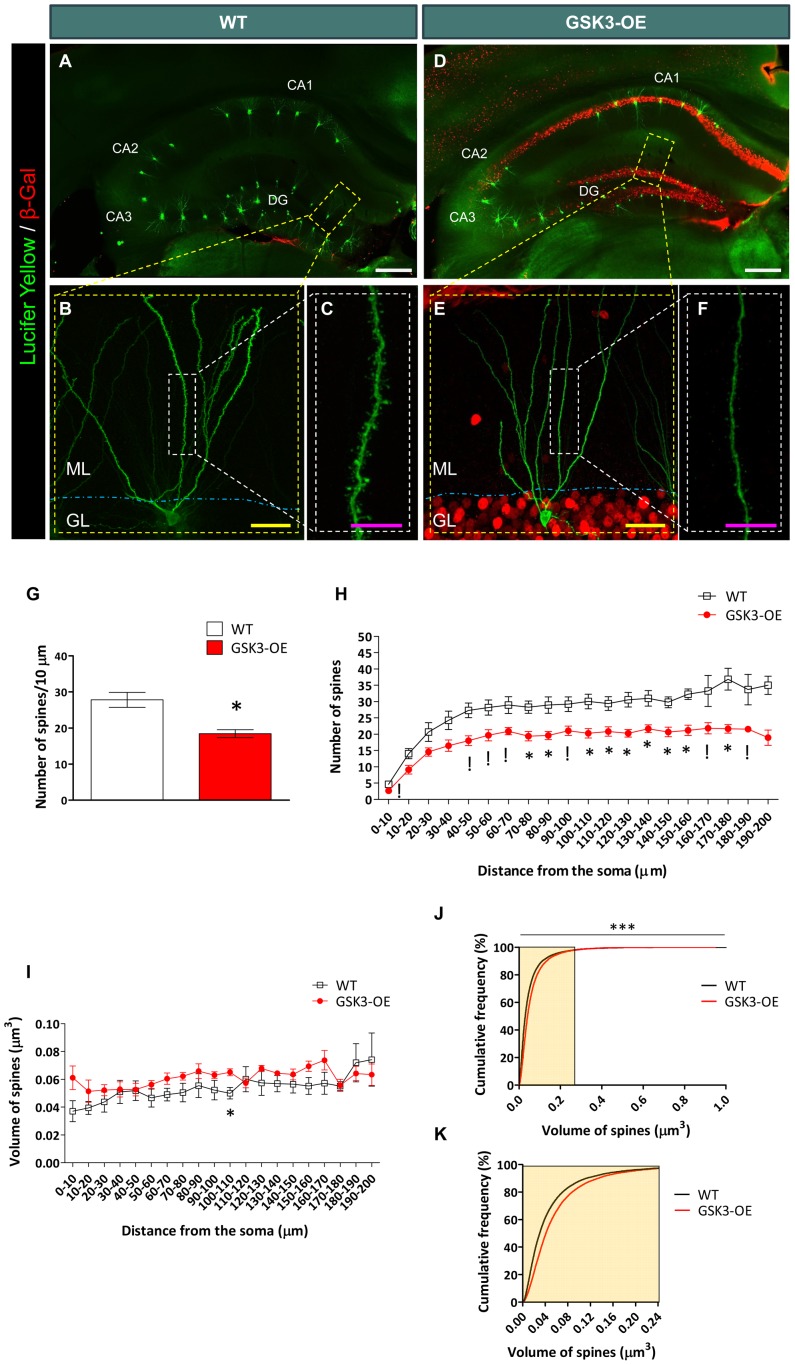
**GSK-3β overexpression alters the number of dendritic spines in developmentally generated granule neurons *in vivo.*** **(A–F)** Representative images of granule neurons (labeled with Lucifer yellow) from WT **(A**–**C)** and GSK-3β-overexpressing (GSK3-OE) **(D–F)** mice. In **(A,** **D)**, tile-scan acquisitions showing the whole hippocampus can be observed. It should be noted that the injections were performed only in granule neurons located in the outer border of the granule layer, in order to ensure that they were generated during development. **(B,E)** Single neurons labeled with Lucifer yellow, as well as high power magnifications (showing their spines (**C**,**F**, respectively). **(G)** Quantification of the average density of spines in WT and GSK3-OE granule neurons. **(H)** Quantification of spine density along the dendritic tree of WT and GSK3-OE granule neurons. GSK-3β overexpression led to a reduced number of spines in almost the entire dendritic tree of these neurons. **(I)** Quantification of the spine volume of WT and GSK3-OE granule neurons along their dendritic trees. **(J)** Representation of the cumulative frequency distribution of the spine size in WT and GSK3-OE granule neurons. A more detailed representation of the frequency distribution of the smallest spines can be observed in **K**. GSK-3β overexpression slightly alters the distribution of spine sizes, although no regional differences in this parameter were observed along the dendritic tree of granule neurons. In **(G**–**I)** graphs represent mean ± SEM; *n* = 4 mice per genotype; ^!^0.1 > *p* ≥ 0.05; ^∗^0.05 > *p* ≥ 0.01 (Mann–Whitney’s *U* test). In **(J)** ^∗∗∗^*p* < 0.001 (Kolmogorov–Smirnov test). DG: dentate gyrus. ML, Molecular layer. GL, Granule layer. White scale bar: 150 μm. Yellow scale bar: 50 μm. Purple scale bar: 10 μm.

In order to determine whether GSK-3β overexpression alters the number of spines in developmentally generated granule neurons, we performed intracellular injections of Lucifer yellow in granule neurons located in the outer edge of the GL of the DG of WT (**Figures 1A,C**) and GSK3-OE mice (**Figures 1D–F**). Given the developmental migratory pattern of these cells, those located in the two outer thirds of this layer have a developmental origin (Kempermann and Gage, 2000). In order to rule out any putative confounding effect caused by altered migration of adult-born granule neurons, the migration of retrovirally labeled 10-week-old newborn granule neurons was measured in GSK3-OE and WT mice (**Supplementary Figure S1**). As can be observed, no differences in the migratory pattern of fully mature newborn granule cells can be observed at this cell age, thus confirming the developmental origin of the neurons located at the outermost position of the GL.

Double immunohistochemistry for Lucifer Yellow or GFP and GSK-3β overexpression reporter (β-Galactosidase) was performed in order to check GSK-3β overexpression in each cell analyzed. No β-Galactosidase^+^ cells were found in WT mice, as previously reported (Llorens-Martin et al., 2013, 2016). GSK-3β overexpression decreased the density of spines in developmentally generated granule neurons (*U* = 0; *p* = 0.029) (**Figure 1G**), and this effect took place along their whole dendritic tree (**Figure 1H** and Supplementary Table S1). However, no marked differences were found in the size of these spines in GSK3-OE mice as compared to WT ones (Supplementary Table S2 and **Figure 1I**). Nevertheless, examination of the cumulative probability distribution of the spines grouped by size revealed that GSK-3β overexpression caused a slight alteration in the size distribution of these structures, the smallest spines being less abundant in GSK3-OE mice than in their WT counterparts (K–S *Z* = 14.258; *p* < 0.001) (**Figures 1J,K**).

### GSK-3β Overexpression Induces Subtle Morphological Alterations in the Spines of Developmentally Generated Granule Neurons

**FIGURE 2:**
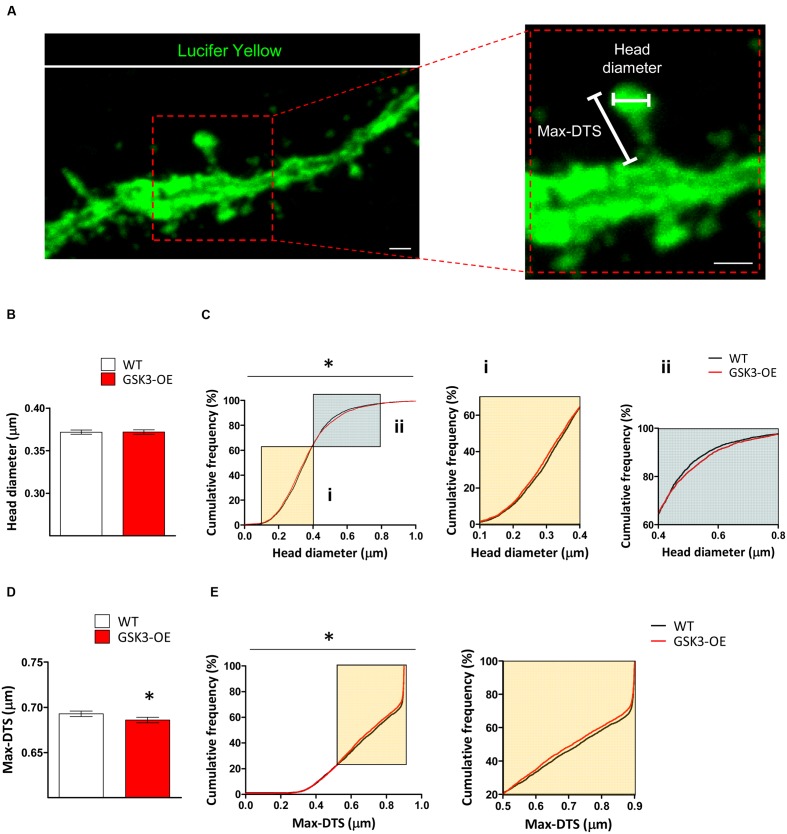
**GSK-3β overexpression results in subtle alterations in the spine morphology of developmentally generated granule neurons.** **(A)** Representative image of a Lucifer yellow-labeled granule neuron showing the parameters used to analyze the morphology of the dendritic spines (head diameter and Max-DTS). **(B–E)** Quantification of the head diameter **(B,C)** and Max-DTS **(D,E)** of dendritic spines in WT and GSK-3β-overexpressing (GSK3-OE) granule neurons. GSK-3β overexpression alters the cumulative frequency distribution of dendritic spine head diameter **(Ci,** **ii)**. In addition, it reduced dendritic spine Max-DTS **(D)** as well as the cumulative frequency distribution of this parameter **(E)**. In **(B,D)**, mean ± SEM is represented; *n* = 4 mice per genotype; ^∗^0.05 > *p* ≥ 0.01 (Mann–Whitney’s *U* test). In **(C,E)**, cumulative frequency distributions are represented; *n* = 4 mice per genotype; ^∗^0.05 > *p* ≥ 0.01 (Kolmogorov–Smirnov test). Scale bars: 1 μm.

In the light of the alterations found in the distribution of spine sizes in GSK3-OE mice, we aimed to further characterize the effects of GSK-3β overexpression on the morphology of these structures. For this purpose, we analyzed both the head spine diameter and an approximate measure of the spine length (the Max-DTS) in Lucifer yellow-labeled developmentally generated granule neurons of WT and GSK3-OE mice (**Figure 2A**). GSK-3β overexpression did not alter the mean head spine diameter (**Figure 2B**). However, examination of the cumulative frequency distribution of this parameter revealed the existence of two distinct populations of spines altered in opposite directions (K–S *Z* = 1.493; *p* = 0.023). Thus, in GSK3-OE neurons those spines with small head diameter became smaller (**Figure 2Ci**), and conversely those spines with big head diameter became bigger (**Figure 2Cii**). On the other hand, GSK-3β overexpression promoted a general decrease in the spine Max-DTS (*U* = 9154017; *p* = 0.020) (**Figure 2D**), being the cumulative frequency distribution of this parameter also altered in the same direction (K–S *Z* = 1.539; *p* = 0.018) (**Figure 2E**). These results together are indicative of a subtle remodeling of the spine morphology in developmentally generated granule neurons promoted by GSK-3β overexpression.

### GSK-3β Overexpression Differently Affects the Morphology of the Distinct Types of Dendritic Spines in Developmentally Generated Granule Neurons

**FIGURE 3:**
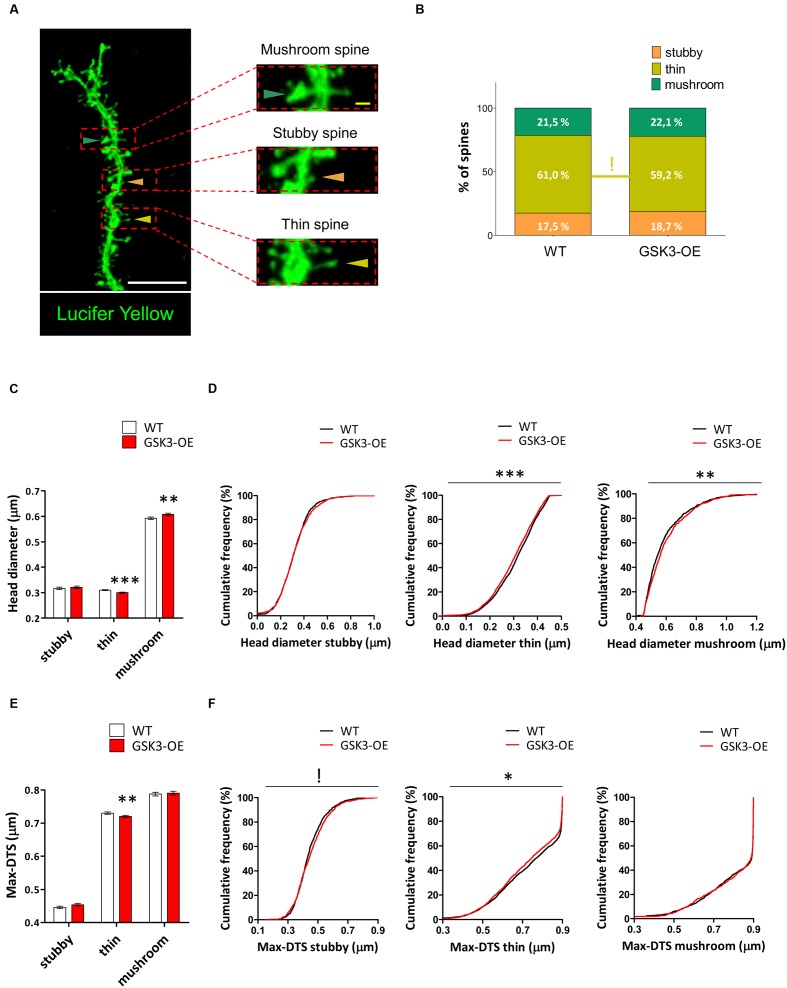
**GSK-3β overexpression differently affects the morphology of the distinct types of dendritic spines in developmentally generated granule neurons.** **(A)** Representative image of a Lucifer yellow-labeled granule neuron showing three distinct spine morphologies: stubby (orange triangles), thin (yellow triangles), and mushroom (blue triangles). **(B)** Quantification of the percentages of stubby, thin, and mushroom spines in WT and GSK-3β-overexpressing (GSK3-OE) granule neurons. GSK-3β overexpression did not affect the proportion of the different types of spine, although a trend towards a reduction in the percentage of thin spines can be observed in GSK3-OE granule neurons. **(C–F)** Quantification of the head diameter **(C,D)** and Max-DTS **(E,F)** of stubby, thin, and mushroom spines in WT and GSK3-OE granule neurons. GSK-3β overexpression led to subtle alterations in the morphology of the thin and mushroom spines. In **(B)** ^!^0.1 > *p* ≥ 0.05 (χ^2^ test). In **(C,E)** mean ± SEM is shown in the graphs; *n* = 4 mice per genotype; ^∗∗^0.01 > *p* ≥ 0.001, ^∗∗∗^*p* < 0.001 (Mann–Whitney’s U test). In **(D,F)** cumulative frequency distributions are shown in the graphs; *n* = 4 mice per genotype; ^!^0.1 > *p* ≥ 0.05, ^∗^0.05 > *p* ≥ 0.01, ^∗∗^0.01 > *p* ≥ 0.001, ^∗∗∗^*p* < 0.001 (Kolmogorov–Smirnov test). White scale bar: 5 μm. Yellow scale bar: 0.5 μm.

Given the intrinsic variability of the spine morphology, we next classified the spines of Lucifer yellow-labeled developmentally generated granule neurons of WT and GSK3-OE mice into three categories (stubby, thin and mushroom) (**Figure 3A**) and analyzed the proportion and morphology of each type of spine. GSK-3β overexpression did not lead to drastic alterations in the percentages of the different types of spines (**Figure 3B**). However, the percentage of thin spines showed a slight reduction in GSK3-OE mice (χ2 = 3.148; *p* = 0.08). In addition, both the head diameter (*U* = 3186547; *p* < 0.001) and the Max-DTS (*U* = 3236117; *p* = 0.002) of thin spines were reduced in these mice. Interestingly, the cumulative frequency distributions of these parameters were also altered (head diameter K–S *Z* = 2.102; *p* < 0.001; Max-DTS K–S *Z* = 1.620; *p* = 0.011). In contrast, the head diameter of mushroom spines was increased (*U* = 417358; *p* = 0.008), being its cumulative frequency distribution also altered in the same direction (K–S *Z* = 1.718; *p* = 0.005). However, no changes in the Max-DTS of this spine subtype were found (**Figures 3C–F**).

## Discussion

The DG is the only hippocampal region in which adult neurogenesis occurs. Due to the continuous addition of newly generated synaptic elements to the preexisting network, the DG has an extraordinarily high degree of plasticity and, consequently, this structure is involved in unique aspects of hippocampus-dependent learning, such as pattern separation (Sahay et al., 2011). The coexistence of mature — either newborn or developmentally generated — and immature elements confers the DG enormous functional complexity, which remains elusive. Thus, the DG should be considered a structure subjected to continuous remodeling and transformation, and affected by plastic changes that only occur in restricted regions of the brain during adulthood. Therefore, how mature and immature elements interact in physiological and pathological conditions is an opened question that should be investigated. In this regard, newborn and developmentally generated granule neurons share some maturational aspects but differ radically in others (Nakashiba et al., 2012). These two types of neurons have markedly different morphologies, which are probably related to the distinct positioning of these two cell types within the granule cell layer. Here we have demonstrated that mature newborn granule neurons (generated at two months of animal age) do not differ in their positioning within the GL between genotypes. Given that 10-week-old newborn neurons were analyzed at 4–5 months of age, that the average migration of these fully mature cells is about 20 μm, and that only those cells located at the outermost position of the GL were included in this study, it is possible to ensure the developmental origin of the cells analyzed.

While the classical morphology of the newborn granule neurons includes the presence of a single and long primary apical dendrite that emerges from the soma and is extensively branched in the molecular layer [namely “Y-shape” (Llorens-Martin et al., 2015b)], the developmentally generated granule neurons usually present several primary apical dendrites, which are shorter than those of newborn neurons, and the “Y-shape” can rarely be appreciated (Llorens-Martin et al., 2015b). Although electrophysiological recordings demonstrate that after completion of the maturational period (approximately 8 weeks) a newborn granule neuron is functionally indistinguishable from its developmentally generated counterpart (Song et al., 2005; Zhao et al., 2006; Bischofberger, 2007), it is reasonable to assume that a series of both cooperative and competitive events occurs between mature and maturing neurons during the different stages of development. In fact, it has been postulated that mature and immature spines compete to establish synaptic contacts with axonal terminals of the perforant pathway (Toni and Sultan, 2011; Krzisch et al., 2015). It is thought that the spines of newborn neurons replace those of mature, developmentally generated granule neurons, in order to allow the functional selection of newly generated neurons for survival (Bosch et al., 2015; Krzisch et al., 2015). Given that mature synaptic connections must be plastic enough to allow their replacement by a new synaptic connection, this process is highly demanding in terms of plasticity. In line with this, GSK-3β is a key regulator of synaptic plasticity (Arendt, 2003; Peineau et al., 2008; Bradley et al., 2012). In fact, it is known that long-term depression (LTD) inhibits LTP through the activation of GSK-3β (Peineau et al., 2007). Thus, an increase in GSK-3β activity results in impaired LTP generation in the hippocampus (Hooper et al., 2007). Although the mechanism by which GSK-3β inhibits LTP generation remains to be fully elucidated, it is known that GSK-3β triggers the endocytosis of the AMPA receptor GluR1 subunit, thereby leading to the elimination of the synapse (Peineau et al., 2008). Consistent with our previous results in newborn granule neurons (Llorens-Martin et al., 2013, 2016), here we demonstrate that GSK-3β overexpression reduces the number of spines in developmentally generated granule neurons. This observation reflects impairment of connectivity in both newborn and developmentally generated granule neurons. Thus, the active elimination of pre-existing synapses emerges as a mechanism by which the dysregulation of GSK-3β activity may result in a net reduction of spines. However, this phenomenon could also be explained by a decrease in the incorporation of new synapses caused by downstream substrates of GSK-3β. Among these, the microtubule-associated protein Tau is considered to be pivotal in neurodegenerative diseases (Takashima et al., 1993). Tau plays a central role in regulating the stability of both microtubules and postsynaptic densities (Ittner et al., 2010; Pallas-Bazarra et al., 2016). In line with this, Tau phosphorylation by GSK-3β has been proposed as a mechanism by which the dysregulation of the activity of this kinase decreases the incorporation of new spines in newborn granule neurons (Llorens-Martin et al., 2016).

It is important to note that the distinct morphological types of spines have been differentially related to the plasticity of synaptic connections. For instance, thin spines are believed to be the most plastic type of spine (McGilvray, 2016). In line with the aforementioned notion, among the different types of spines, thin ones account for the highest proportion in granule neurons. In particular, a decrease in the proportion of thin spines has been reported in Alzheimer’s disease (AD) patients (McGilvray, 2016). In addition, we have previously demonstrated that GSK-3β overexpression, which is also related to AD (Shiurba et al., 1996; Leroy et al., 2002; Jope and Johnson, 2004), causes a decrease in the percentage of both thin and mushroom spines in newborn granule neurons (Llorens-Martin et al., 2016). In the present study, we report that GSK-3β overexpression tends to lead to a decrease in the proportion and size of thin spines, together with a subtle increase in the head volume of mushroom spines in developmentally generated granule neurons. On the other hand, a decrease in the volume of postsynaptic clusters (Llorens-Martin et al., 2013), together with a decrease in the percentage of mushroom spines (Llorens-Martin et al., 2016), has been observed in GSK3-overexpressing newborn granule neurons. Although GSK-3β decreases the density of spines both in newborn (Llorens-Martin et al., 2016) and developmentally generated granule neurons (as our present data reveal), a differential effect on the percentages and morphometric parameters of the different types of spines seem to occur in these two cell populations. Interestingly, it has recently been demonstrated that the synaptic connectivity of mature granule neurons negatively affects the functional integration of newly generated ones (McAvoy et al., 2016). Thus, GSK-3β overexpression increased the head area of mushroom spines and reduced that of thin ones. According to the recent data published by McAvoy et al., the stability of these synaptic connections may be detrimental for the addition of newly generated synaptic contacts. Such contacts ultimately determine the resulting plasticity of a given synaptic network, which is necessary for learning and for the acquisition of new memories (Sahay et al., 2011)

Thus, in line with our previous results, we propose that GSK-3β overexpression in the hippocampus leads to a deficit in synaptic plasticity (Peineau et al., 2008) and impairment of the functional maturation of newborn granule neurons (Llorens-Martin et al., 2013, 2016). Furthermore, as we demonstrate here, GSK-3β overexpression in this brain region triggers a series of alterations in the spines of the oldest elements present in the DG, namely developmentally generated mature granule neurons. Further studies are required to unravel the causal relationship between the aforementioned phenomena, as well as the deleterious consequences of the dysregulation of GSK-3β activity on the intriguing coupling between plasticity and stability that drives DG function.

## Ethics Statement

All applicable international, national, and/or institutional guidelines for the care and use of animals were followed. All procedures performed in studies involving animals were in accordance with European Community Guidelines (directive 86/609/EEC) and were handled following European and local animal care protocols.

## Author Contributions

JA, JD, and MLL-M conceived and designed the experiments; NP-B and AK performed the experiments; AK acquired confocal images; NP-B and AK analyzed the data; JA, JD, and MLL-M got funding; MLL-M, JA, NP-B, and JD wrote the manuscript. All the authors revised and approved the final form of the manuscript.

## Conflict of Interest Statement

The authors declare that the research was conducted in the absence of any commercial or financial relationships that could be construed as a potential conflict of interest.

## Supplementary Material

**FIGURE S1 | GSK-3β overexpression does not alter fully mature newborn neuron migration.** **(A)** Representative image of retrovirally labeled 10 week old newborn granule neurons in the dentate gyrus. **(B)** Quantification of the distance between the hilar border of the subgranular zone and the center of the cell nucleus in WT and GSK3-OE newborn neurons. GSK-3β overexpression does not alter the migration of fully mature newborn granule neurons. Graph represent mean ± SEM; *n* = 100 cells per genotype. Scale bar: 50 mm. ML, molecular layer; GL, granular layer; H, hilus.
