# Supplementary figures and images for: GSK-3β Overexpression Alters the Dendritic Spines of Developmentally Generated Granule Neurons in the Mouse Hippocampal Dentate Gyrus
Source: Front Neuroanat. 2017 Mar 10;11:18. doi: 10.3389/fnana.2017.00018 (PMC5344922; doi:10.3389/fnana.2017.00018)

**A**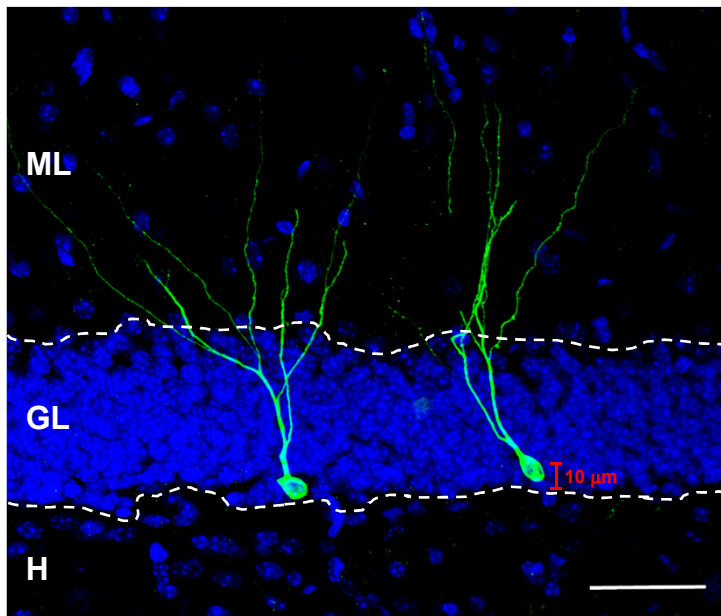**B**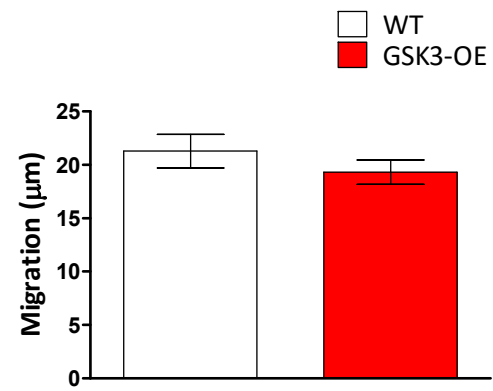

Supplement: Supplementary file 1 [file Image_1.pdf]
