# Supplementary material for: GSK-3β Overexpression Alters the Dendritic Spines of Developmentally Generated Granule Neurons in the Mouse Hippocampal Dentate Gyrus
Source: Front Neuroanat. 2017 Mar 10;11:18. doi: 10.3389/fnana.2017.00018 (PMC5344922; doi:10.3389/fnana.2017.00018)
Supplement: Supplementary file 3 [file Table_2.docx]

| **Distance from the soma (µm)** | **Mann-Whitney U** | **p-value** |
| --- | --- | --- |
| 0 - 10 | 13,0 | 0,200 |
| 10 - 20 | 12,0 | 0,343 |
| 20-30 | 11,0 | 0,486 |
| 30 - 40 | 9,0 | 1,000 |
| 40 - 50 | 8,0 | 1,000 |
| 50 - 60 | 13,0 | 0,200 |
| 60 - 70 | 14,0 | 0,114 |
| 70 - 80 | 14,0 | 0,114 |
| 80 - 90 | 11,0 | 0,486 |
| 90 - 100 | 13,0 | 0,200 |
| 100 - 110 | 16,0 | 0,029 |
| 110 - 120 | 7,0 | 0,886 |
| 120 - 130 | 10,0 | 0,686 |
| 130 - 140 | 12,0 | 0,343 |
| 140 - 150 | 11,0 | 0,486 |
| 150 - 160 | 14,0 | 0,114 |
| 160 - 170 | 12,0 | 0,343 |
| 170 - 180 | 9,0 | 1,000 |
| 180 - 190 | 6,0 | 1,000 |
| 190 - 200 | 5,0 | 0,857 |

**Table S2. Statistical comparisons of the spine volume along the dendritic tree of granule neurons from WT and GSK3-OE mice.** Mann Whitney´s U test; n= 4 mice per genotype.
